# Collective ritual and social support networks in rural South India

**DOI:** 10.1098/rspb.2018.0023

**Published:** 2018-05-23

**Authors:** Eleanor A. Power

**Affiliations:** London School of Economics and Political Science, Houghton Street, London WC2A 2AE, UK

**Keywords:** social networks, collective ritual, cooperation, social cohesion, religion

## Abstract

The scholarship on religion has long argued that collective worship helps foster social cohesion. Despite the pervasiveness of this contention, rigorous quantitative evaluations of it have been surprisingly limited. Here, I draw on network data representing the ties of social support among Hindu residents of a South Indian village to evaluate the association between collective religious ritual and social cohesion. I find that those who partake in collective religious rituals together have a higher probability of having a supportive relationship than those who do not. At the structural level, this corresponds to denser connections among co-participants. At the individual level, participants are more embedded in the local community of co-religionists, but are not disassociating themselves from members of other religious denominations. These patterns hold most strongly for co-participation in the recurrent, low-arousal monthly worships at the temple, and are suggestive for co-participation in the intense and dysphoric ritual acts carried out as part of an annual festival. Together, these findings provide clear empirical evidence of the lasting relationship between collective religious ritual and social cohesion.

## 1. Introduction

The scholarship on religion has long suggested that collective rituals bind participants into a moral community. This is a central point of much of the foundational sociological work on religion [1–3], as well as more recent formulations drawing from economic and evolutionary theory [4–9]. Recent evolutionary explanations of religion have suggested that our species' ‘ultrasociality’—our large-scale societies of unrelated individuals—may be thanks in part to religion's ability to help forge cohesive, cooperative social groups [6,9–11,13]. While some of these accounts focus on religious belief [15–17], many also highlight the role of ritual in fostering this social group [9,11–14]. Collective rituals may evoke innate psychologies that build a sense of affiliation, fusion and kinship among participants as they move in synchrony and experience pain and euphoria together [8]. This sense of camaraderie and kinship, buttressed further by associated religious beliefs, may then help co-participants form into a clear social group. For these accounts of the origins of religion and human ultrasociality, the fact that religions form *groups* of committed, cooperative, like-minded individuals is paramount to religion's influence and ubiquity, in part because it may allow for cultural group selection [6,7,9,18,19].

Despite the common appreciation of the importance of collective ritual, quantitative evidence of the relationship between collective ritual and social cohesion is surprisingly limited. Studying firewalking rituals in Spain and Mauritius, Xygalatas and colleagues [20–22] have found that firewalkers experience elevated feelings of happiness and increased heart rate during and after the ritual, with other onlookers, especially close relatives, experiencing synchronized arousal and sympathetic fatigue. There have been some attempts to document the particular elements of collective ritual that may foster cohesion. Experimental work has found that those who experience pain together play more cooperatively in economic games [23]. Other work has investigated the effect of synchrony of movement and voices on cooperation and bonding, though the results have been somewhat mixed [24–28]. Such studies provide preliminary suggestions that the shared experience of collective rituals may help forge connections between co-participants.

This increased cohesion within the in-group may be made in contradistinction to an out-group. Such ‘parochial altruism’ [29,30] may be particularly acute in religious groups, as many of their beliefs and practices mark them off as distinct from wider society. Along with studies showing greater trust and cooperation among co-religionists [31,32] are others showing that religious identity and participation is associated with out-group hostility [33–36], and that even arbitrary rituals can promote out-group bias [37]. Such findings suggest that collective worship fosters a form of ‘coalitional commitment’ that includes both greater cooperativeness and cohesion with religious peers and also distancing from religious others.

While these studies hint at collective ritual's cohesive effects, they are often only suggestive. Experimental work has largely looked at only fleeting evidence of cohesion in the immediate aftermath of a collective ritual, while survey-based work has often used religious group membership as a proxy for co-participation. The evidence for cohesion, then, is either local and artificial or broad and abstracted. And, the fundamental proposition that a *group* results from collective ritual has not been tackled empirically. Here, I focus on the long-lasting, tangible aspects of cohesion, defining it as the supportive relationships that link people together. To quantify this, I draw on social network data detailing the supportive relationships among Hindu residents of a South Indian village that I call by the pseudonym ‘Tenpaṭṭi’. There, Hindu residents partake in both ‘doctrinal’ (recurrent, low-arousal) rituals when they attend a monthly worship and in ‘imagistic’ (rare but intense, often dysphoric) rituals when they take part in the annual village festival [8,38]. In what follows, I look at the individual, interpersonal, and structural correlates of co-participation in both forms of collective ritual in order to determine whether collective ritual is indeed associated with greater social cohesion.

## 2. Study site

‘Tenpaṭṭi’ is located in the irrigation-fed scrublands near the Vaigai River in Tamil Nadu, India. Most villagers combine manual wage labour with agriculture, growing rice and other crops on small plots of land. Villagers assist one another in many ways, watching each other's children, sharing meals, working in each other's fields, relaying news of job opportunities and pooling their money in microfinance loan groups. Residents represent multiple caste groups and include both Hindu and Catholic faiths (electronic supplementary material, table S1), though this study is limited to the Hindu residents.

Hindu religious activity in Tenpaṭṭi centres around the temple for the goddess Māriyamman, who ensures the wellbeing of the village and its residents. Each month, many Hindu residents attend a worship at her temple (the *paurnami p*ū*jai*), making offerings and prayers, and socializing with others in attendance. Each summer, the village organizes a week-long festival for the goddess. The crucial event is the carrying of the *muḷaipp*ā*ri*, pots with bright green sprouts representing the village's vitality. The climax of the festival, though, is the procession of vow-takers. During the year, men and women make vows to the goddess in hopes that she will intervene in their lives (help having a child, finding work, etc.). Fulfilling such vows requires many days of fasting, during which vow-takers abide by various restrictions, including wearing particular clothes, eating only one (vegetarian) meal a day, abstaining from sex and eschewing alcohol and nicotine. At the culmination of the festival, the vow-takers fulfil their vows, performing acts such as walking across a bed of hot coals, piercing their cheeks with a spear, carrying flaming firepots or pouring milk over the image of the goddess. Collective rituals for the goddess are, therefore, of three types: the ‘doctrinal’ monthly worship, the ‘imagistic’ procession of vow-takers in the annual festival and the low-arousal *muḷaipp*ā*ri* procession in the annual festival. Observing participation in such rituals appears to influence how others perceive a person [39] and how they relate to her [40]; here I ask what role *co*-participation plays in those relationships.

Following the scholarship on religion, co-participation in both the monthly worship and in the annual Māriyamman festival should bind people together and foster a religious community. Specifically, I derive four predictions. (1a) Co-participation in the most intense, ‘imagistic’ religious act of the annual festival—the procession of vow-takers—will be associated with an increased probability of a tie between participants. (1b) Co-attendance at the ‘doctrinal’ monthly worship will be associated with an increased probability of a tie between participants. (2) The network of collective ritual participants will be more cohesive than the network of all Hindu residents, as measured by an excess number of social support ties, and higher density, transitivity and reciprocity. And, if co-participants do indeed identify and associate more strongly with their religious peers, then they may consequently dissociate from religious others. So, (3) participants will be less likely to rely on individuals of different religious denominations for support. It is important to note that the data used to test these predictions are cross-sectional, so I will not be able to make claims about the direction of causality.

## 3. Material and methods

### (a) Social support networks

**Figure 1.**
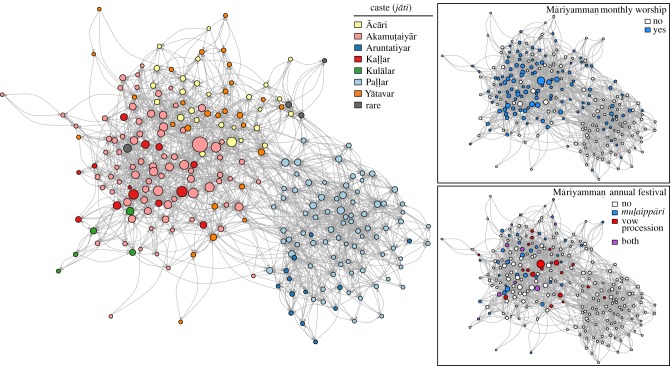
The Tenpaṭṭi Hindu support network (*N* = 248), with colours showing (left) caste, (right top) those who attend the monthly worship, and (right bottom) those who participated in various ways in the 2012 Māriyamman festival. Edges are directed, with an arrow directed from the person requesting support to the person providing it. Node location is determined by the Fruchterman–Reingold algorithm.

The social support network is constructed from a survey conducted with the adult residents of Tenpaṭṭi (*N* = 362, 98%), here limited to the Hindu residents (*N* = 248, 97%). All interviewees provided oral consent. The survey consisted of 12 questions asking interviewees to name the people whom they rely upon for various types of support. The questions were meant to elicit personal bonds of affection and guidance (conversation partners, close friends, advice, important issues), relations of instrumental aid (borrowing items, running errands, lending cash, babysitting, help in finding work, loans), and support accessed rarely but crucially (aid when there is some problem, help in navigating bureaucracy). On average, interviewees named 22 individuals as providing them with some kind of support, of which 13 were other adult Hindu residents. Basic demographic information (age, gender, place of residence, employment, caste, religion) was gathered about each named person. Residents also identified their kin, represented here as a network of close kin (including parents/children, siblings and spouses). Household locations were determined with a GPS unit and satellite imagery, with distances between households calculated in ArcGIS v. 10.0. The relationships elicited through the survey can be used to create a network representing the flows of support among Hindu villagers (figure 1, see electronic supplementary material, section S1.2 for further details).

### (b) Collective worship

All individuals who participate in a ritual are considered to be co-participants (see electronic supplementary material, section S1.1 for further details). Participation in the 2012 Tenpaṭṭi Māriyamman festival (held 9 months prior to the survey) is determined by records kept by the organizing committee and video footage. The two main events of the festival are analysed: the procession of vow-takers and the *muḷaipp*ā*ri* procession. Of those Hindus included in the social support network, 28 participated in the procession of vow-takers and 43 participated in the *muḷaipp*ā*ri* procession. Attendance at the monthly worship at the Māriyamman temple is based on self-reports and lists made with primary informants. One hundred and twenty-three (50%) of the adult Hindu residents (who completed the survey) attend this collective ritual.

### (c) Statistical analyses

The networks are constructed in R v. 3.4.0 [41] using the igraph package [42] and analysed with the statnet suite of packages [43]. For Predictions 1a and 1b, I model the social support ties using exponential random graph models (ERGMs). ERGMs are statistical models that predict the probability of an tie, given node, edge and structural covariates [44–46]. In addition to other covariates (e.g. age, caste, gender homophily, distance between households, shared partners), I include a variable for whether each set of individuals participated together in each act of the 2012 Māriyamman festival (Prediction 1a) or worship together each month (Prediction 1b). For Prediction 2, I look at the structural correlates of collective ritual, calculating measures of network cohesion for each co-participant subgraph (the subset of the network including only participants and ties between them). I calculate the network density (the percent of all possible ties in a network that are actually observed), transitivity (also known as the global clustering coefficient or ‘the fraction of transitive triples’, how many of the triplets of nodes in a network are closed, forming a triangle) and reciprocity (the probability of a tie existing in one direction, given a tie in the other) [47]. I additionally calculate the ‘excess edges’ of each subgraph: the number of edges in the subgraph minus the number expected in a randomization of the edges that preserves the in- and out-degree of each node (see electronic supplementary material, section S3 for further details). While density and ‘excess edges’ give a sense of the overall connectedness of the network, reciprocity and transitivity measure the degree to which those connections are shaped by reciprocal relationships or common partners. To determine whether the differences in network cohesion are significantly greater than expected, for each co-participation subgraph I generate 10 000 networks of the same size by selecting nodes randomly from the set of possible participants, retaining the actual ties between those nodes, and calculating the cohesion measures for each. From this, I assess where the observed network cohesion measure falls within the distribution of possible values. Finally, for Prediction 3, I look at participants' relationships beyond the Hindu community by modelling the proportion of a person's support ties to members of other religious denominations using a binomial regression with demographic covariates and individual random effects. Models are performed using the map2stan function in the rethinking package [48].

## 4. Results

### (a) Collective rituals increase the probability of a support tie

For festival participants, only those who participate together in the ‘imagistic’ procession of vow-takers have an increased probability of a support tie between them (table 1; electronic supplementary material, table S9 and figure S3a), being 1.3 times as likely to have a tie as two people who do not participate together, though the significance of this effect is marginal (*p*-value of 0.07, 90% confidence interval of 1.02 to 1.72). Co-attendance at the ‘doctrinal’ monthly worship significantly increases the probability of a support tie (table 1; electronic supplementary material, table S10 and figure S3b), with two people being 1.3 times as likely to have a tie if they both attend than if they do not (90% confidence interval of 1.22 to 1.42). When all measures of co-participation, as well as terms for the individual effect of a person's religious action [40], are included in the same model (electronic supplementary material, tables S11 and S12, figure S3c), the effect of co-attendance at the monthly worship stays largely unchanged, while the effect of vow procession co-participation is diminished and is not significant.

**Table 1.** ERGM results for (*a*) co-participation in the annual festival and (*b*) co-participation in the monthly worship. (Full models in the electronic supplementary material.)

	estimate	s.e.	odds ratio	*p*-value
(*a*) vow procession (no = 0)	0.282	0.158	1.326	0.0739
*muḷaippāri* procession (no = 0)	0.130	0.111	1.138	0.2449
(*b*) monthly worship (no = 0)	0.274	0.048	1.315	<0.0001

### (b) Co-participants form denser subgraphs

The predicted increases in graph density, transitivity, reciprocity and excess edges are generally found, though the increases are not always greater than would otherwise be expected (table 2 and electronic supplementary material, table S13 for *p*-values with Bonferroni correction). Co-participants, whether in the festival or in the monthly worship, form a substantially denser subgraph with more excess edges than would be expected. The subgraph of monthly worshippers is 1.6 times as dense and the subgraph of festival participants 1.7 times as dense as the overall Hindu network. Those who are part of the procession of vow-takers are connected together yet more densely (the subgraph of vow-takers is 1.3 times as dense as that of all festival participants, and 2.1 times as dense as that of all resident Hindus) and with more edges, though not more than would be otherwise expected when compared to all festival participants. In contrast, the increases for transitivity and reciprocity are generally not more than would be expected.

**Table 2.** Measures of cohesion (excess edges, density, transitivity, and reciprocity) of the network subgraphs for each type of co-participation.

	excess edges		density		transitivity		reciprocity	
	value	*p*-value	value	*p*-value	value	*p*-value	value	*p*-value
all Hindu			0.027		0.208		0.366	
monthly worship	95.047	<0.0001	0.042	<0.0001	0.208	0.4841	0.413	0.0376
annual festival	50.045	<0.0001	0.045	<0.0001	0.272	0.1038	0.429	0.1369
vow procession^a^	2.956	0.1610	0.058	0.0774	0.231	0.5529	0.364	0.7206

^a^The reference group here is all festival participants.

### (c) Participants are not biased against the religious out-group

Overall, 9.7% of the support ties reported by the Hindu residents of Tenpaṭṭi are to people of different faiths (primarily Catholics), with 62% of people naming at least one alter of another religion. The binomial regressions show no evidence for greater parochialism among ritual participants: individuals who attend the monthly worship or partake in either collective ritual at the annual festival are no more or less likely to name alters of other religious faiths (table 3, full results with covariates in electronic supplementary material, table S14 and figure S4).

**Table 3.** Results of binomial regressions modeling people's ties to alters of other religious denominations, including whether they (*a*) participate in the monthly worship or (*b*) participate in the annual festival. (Full models in the electronic supplementary material.)

	estimate	s.d.	95% HPDI
(*a*) monthly worship (no = 0)	−0.024	0.184	(−0.373, 0.350)
(*b*) *muḷaippāri* procession (no = 0)	−0.001	0.243	(−0.490, 0.465)
vow procession (no = 0)	0.011	0.248	(−0.471, 0.510)

## 5. Discussion

The analyses presented here evaluate the individual, interpersonal and structural correlates of collective religious ritual. Collective ritual is indeed associated with an increased probability of a supportive tie between participants. This holds most strongly for co-attendance at the recurrent, ‘doctrinal’ monthly worship, and is suggestive and trending in the expected direction for co-participation in the rare and intense ‘imagistic’ procession of vow-takers at the annual festival. The cohesive correlates of collective ritual are apparent not only at the interpersonal level, but also at the group level, with co-participation in both the monthly worship and annual festival being associated with substantial increases in network density. That increased cohesion, however, is not coming at the cost of forgone relationships with members of other religious denominations. Importantly, these are not fleeting relationships, but ones that are long-standing and crucial to daily life.

The intense commingling entailed in collective rituals that social scientists have long described is here evaluated quantitatively. In the foundational accounts of Durkheim [1] and others, collective rituals are seen as helping people recognize a commonality with others, and so serve to affectively and practically link the individual to the collective. In Hinduism, that collective is generally not a salient category: there is no ‘congregation’ associated with each temple. As such, the association found here between collective ritual and social cohesion is unlikely to result from a preexisting sense of membership to a religious group. In Hinduism, too, collective rituals take many forms, allowing a study of their differential association with measures of cohesion.

The ERG model results are complemented by the structural analyses, which show greater network density, but not greater transitivity or direct reciprocity, among ritual co-participants. In other words, the communities of co-participants show an increase in ties between them, but not a clustering of those ties between already-connected individuals. This suggests a more general, unstructured condensation of social relationships, primarily between people who may not otherwise have been connected, perhaps suggesting a lingering signature of Turner's unstructured ‘communitas’ [2]. The support network is strongly structured by various forms of social entanglement, as seen in the strong effects of kinship, caste homophily, reciprocity and shared partners in the ERGMs. With the Hindu community divided into different castes and neighbourhoods, collective rituals are one of the few spaces in which these socially distant individuals come together. This diffuse increase in supportive relationships is also potentially suggestive of generalized or upstream reciprocity among co-participants [49–51].

Throughout, I have contrasted ‘imagistic’ and ‘doctrinal’ rituals, referencing Whitehouse's delineation of two ‘modes of religiosity’ [38]. Whitehouse & Lanman [8] have recently suggested that these two modes result in different forms of social cohesion, with ‘imagistic’ rituals leading to identity fusion and a willingness to sacrifice for co-participants and ‘doctrinal’ rituals fostering a sense of shared group identification promoting trust and cooperativeness. Without data on individuals' perceptions of their religious communities and identities, I am unable to directly test these predictions, but I find evidence for the cohesive correlates of both modes of ritual practice. It is telling that it is only the truly ‘imagistic’ ritual of the festival, the intense, dysphoric procession of the vow-takers, that is (marginally) associated with greater social cohesion and not the relatively low-arousal *muḷaipp*ā*ri* procession. Still, when additional model terms representing the direct effect of an individual's religious acts are included, the effect of vow procession co-participation is diminished, while the effect of monthly worship co-participation remains largely unaltered. This suggests that it is not so much the shared experience as the shared observation of other co-participants that explains the increased probability of a tie among the vow-takers. While work on ‘imagistic’, dysphoric rituals has highlighted how it may lead to an immediate experience of ‘collective effervescence’ [20], long-lasting tangible relationships may be more strongly associated with recurrent ‘doctrinal’ rituals. Notably, these two modes are not alternatives; many Hindus partake in both ritual modalities, with the two reinforcing one another.

Importantly, participants in collective rituals appear to be benefitting from greater embeddedness and close ties with co-religionists *without* sacrificing relationships beyond that community: whether a person participates in collective rituals does not impact her likelihood of having supportive partners of other religious denominations. While Hinduism's syncretism and lack of obviously defined religious groups may make parochialism less likely, such boundary-crossing relationships may generally be more common than might be thought. In the USA, for example, the evidence for a consistent out-group bias among the religious is present, but weak [34], and most religious Americans have non-religious peers [52]. Similarly, while the religiously affiliated may report being less trusting of people of other faiths, this may be due to other important covariates [53], and is not necessarily accompanied by behavioural shifts [31]. These findings echo the somewhat equivocal evidence for parochial altruism more generally [54]. Being able to strengthen in-group ties without sacrificing out-group relationships means that the Hindu ritual participants are able to have both ‘bonding’ and ‘bridging’ social capital [55]. A number of studies have highlighted the ‘bonding’ social capital that relationships with co-religionists can provide, demonstrating participants' access to crucial material aid [56–59] and their improved well-being [60–62]. There is also value, though, in ‘bridging’ social capital, particularly weaker ties to those who have access to different knowledge and resources [63–66]. Maintaining relationships with members of other religions may then be a welcome complement to the benefits of embeddedness within the religious in-group [56,67].

While these analyses are highly suggestive of the impact of collective worship on supportive relationships, I cannot make strong claims about the direction of causality. I would suggest, however, that the effects found in the analyses here are the result of causal arrows going both ways. People are drawn into collective rituals by their peers [68,69], and while there, their relationships with other participants can further strengthen, making both future support and future attendance more likely. There is a cycle of ever-increasing socioreligious entanglement (both interpersonally, and, as the network cohesion measures suggests, structurally, too) among those who worship together, in whatever ways. Longitudinal network data will hopefully help to establish the relative importance of these two mechanisms drawing people together.

## 6. Conclusion

Accounts of the cohesive effects of religion often talk of the creation of a religious group, a moral community. For cultural group selection to play a role in the evolution of religion as many suggest [6,7,9,18,19], such groups are an essential element. However, the existence of a religious group is often presumed, as is the role of collective ritual in forming that group. Here, I have attempted to empirically evaluate whether co-participants can actually be seen as comprising a group, and have found suggestive evidence that they do. This has been facilitated by a network approach, which allows for scale-bridging analyses that can concretely identify the social correlates of collective worship at the individual, interpersonal and structural levels. A network approach forces a clear definition of what the group is and how cohesion will be evaluated. It does not require that groups have hard, clear boundaries, but instead allows for more fluid ideas of belonging, with individuals being part of multiple overlapping and variably defined communities. Empirically and theoretically, it may be better to search for evidence of the condensation of relationships among co-participants, rather than for a ‘group’ per se. Such a shift allows for a concrete but general definition of the ‘group’, and avoids any implicit assumption that all religions follow the Abrahamic pattern of clearly bounded ‘congregations’. Indeed, only be eschewing the presumption of a religious group can its existence be made empirically testable.

This network approach to identifying social cohesion has proved to be particularly important here, as Hindu ritual participants appear to be simultaneously building a strong supportive community while also maintaining relationships beyond it. A network approach can allow for a simultaneous study of how collective ritual can forge bonds within a community, as well as how rituals may shape relationships beyond it. And, rather than speaking abstractly of the broad benefits associated with membership to a religious group, grounding the study in the actual relationships between individuals makes those benefits tangible: the material and immaterial flows of support studied here are clearly of consequence to people's livelihood and wellbeing. By demonstrating collective ritual's cohesive correlates, these analyses will hopefully provide important empirical fodder for further theoretical inquiry into the relationship between collective ritual and social cohesion.

## Supplementary Material

Supplementary Material

## Data accessibility

Those interested in accessing the anonymized data should contact the author. Code to generate the findings is accessible at: https://github.com/eapower/CollectiveRitual.
