# Supplementary material for: Collective ritual and social support networks in rural South India
Source: Proc Biol Sci. 2018 May 23;285(1879):20180023. doi: 10.1098/rspb.2018.0023 (PMC5998092; doi:10.1098/rspb.2018.0023)
Supplement: Supplementary Material [file rspb20180023supp1.pdf]

Electronic Supplementary Material for "Collective Ritual and Social Support Networks in Rural South India" by Eleanor A. Power

S1 Background Information

This paper is based on fieldwork conducted in the village of "Teṉpaṭṭi" since 2011, and draws primarily on a survey done with adult residents in April 2013 and records of participation in the annual festival for the goddess Māriyammaṉ that took place in August 2012. Additional background information on this fieldwork and the village, as well as the neighbouring village of "Aḻakāpuram," can be found in my other publications, most notably the supplementary material for Ref. [40].

"Teṉpaṭṭi" is surrounded by the small agricultural plots of the residents, and scrubby underbrush beyond. It borders a regional highway, which is getting increasingly built up. This provides access to the nearby market towns, and the city of Madurai, roughly an hour away by bus. Most villagers engage in agriculture, either as landowners and/or as agricultural labourers. This is generally supplemented by manual labor and work in the government's "100 Days Work" scheme. With educational attainment rapidly increasing, younger residents are increasingly working in skilled or semi-skilled jobs in nearby towns and cities, and some are now working abroad. The government provides many basic services to villagers, including subsidised food, housing loans, free education and training programs, family planning programs, etc. "Teṉpaṭṭi" has the local government panchayat office, through which such services are dispersed. The village has both elected representatives to the panchayat, and an informal village council, with representatives from each caste community.

Village residents represent a number of different caste and religious denominations (Table S1). All residents are Hindu, except some of the families of the Yātavar caste. These families have been Catholic for many generations, and worship at church in the village or in the nearby town. There are multiple caste (*jāti*) groups in the village, almost exclusively of historically marginalised groups (Backwards and Scheduled Castes, in the terminology of the Indian state). The Scheduled Castes in this village are the Aruntatiyar and Paḷḷar castes. They have endured a long history of discrimination and disenfranchisement, and continue to do so in many ways today, even with government policies of affirmative action. Within the village, their neighbourhood is somewhat set off, and they are still generally economically disadvantaged, compared to their BC peers. Scheduled and Backward caste groups today rally around caste-based political parties and organisations, using a discourse of rights and caste pride, adding a sometimes combative stance to inter- and intra-caste relations in this village and in the region. Still, as the networks show, there are many relationships that span caste divides, including between SC and BC residents.

Table S1: The number of households, residents, and adult residents of Teṉpaṭṭi broken down by caste and religious denomination. Note that 7 of the 255 (3%) of the adult Hindu residents did not complete the social support network survey, and so are excluded from the analyses here.

	Households	Residents	Adults
**Caste (*jāti*)**			
Ācāri	13	42	27
Akamuṭaiyār	35	111	81
Aruntatiyar	7	17	14
Kaḷḷar	6	19	13
Kulālar	2	7	5
Nāyakkar	1	4	3
Paḷḷar	39	125	81
Veḷḷāḷar	1	3	1
RC Yātavar	48	168	116
Hindu Yātavar	12	39	30
**Reservations**			
Scheduled Castes	46	142	95
Backward Castes	118	393	276
**Religion**			
Hindu	116	367	255
Roman Catholic	48	168	116
**Total**	164	535	371

S1.1 Collective Worship

This paper focuses on the Hindu residents of Teṉpaṭṭi and their religious lives. The village has a number of temples, most notably a temple at the village entrance to the elephant god Piḷḷaiyār, a temple at the opposite village boundary for the guardian deity Aiyaṉār, and the temple for the village goddess Māriyammaṉ located at the centre of the village. Hindu residents visit these and other temples, both within and beyond the village. Many also worship privately in their homes, with small images of the gods. For more details on the Hindu religious practice in the village, see Refs. [39] and [40].

Many, but not all, of the Hindu residents take part in some way in the collective religious rituals at the Māriyammaṉ temple (Table S2). Each month, on the new moon, the temple priest carries out a worship, the *paurṉami pūjai*, attended by many Hindu residents (Table S4). The priest bathes and dresses the image of the goddess, and presents her with various offerings, some of which are eventually given to attendees as sanctified food (*piracātam*). The *pūjai* usually lasts about 45 minutes, with much of that time taken by the priest's preparations. During that time, attendees chat casually and watch the preparations. Once the goddess and her offerings are prepared, attention turns to the goddess, as people take *tarṣaṉ*, the mutual viewing between the deity and the devotee. The priest will perform *ārtti*, presenting a flame to the deity, then bringing the flame and sacred ash for the attendees to take, dabbing some sacred ash and *kuṅkumam* on their foreheads. During this period, some attendees may suddenly become possessed by the goddess, with her power (*ṣakti*) coursing through their bodies. Such possession usually lasts for only a few minutes, with the person pacing with arms outstretched, eyes bulging and tongue flared. Often, possession spontaneously spreads, with the deity's *ṣakti* spreading to multiple people. The priest will sometimes come to calm the possessed, helping the deity leave the person by dabbing sacred ash on her forehead. In a group of perhaps 100 attendees, only a handful would typically become possessed. Most will simply watch the *pūjai*, converse with friends, take *tarṣaṉ*, offer a prayer, and eat some *piracātam* before returning home.

While the monthly *pūjai* is attended almost exclusively by residents, many more people come to take part in the annual festival for the goddess (Table S5). This event is a highlight of the year for the village, entailing months of preparation. The festival itself, occurring in the Tamil month of Āṭi (mid-July to mid-August), is a week-long affair, with daily events culminating with the fulfilment of vows (*nērttikkaṭaṉ*) and the carrying of the *muḷaippāri*. The festival is opened by the raising of a flag at the temple, and is similarly closed by the lowering of the flag. At the flag raising, those people who are intending to fulfil a vow tie a *kāppu* (a piece of cloth containing turmeric and other items) around their wrists and begin a period of fasting (*viratam*) in anticipation of the ritual acts they will complete at the culmination of the festival. (Some vow-fulfillers, particularly those performing larger acts of devotion will have started fasting prior to this event, up to a month prior). This fasting entails a variety of restrictions, including specific attire (red or yellow clothes, no shoes), diet (no meat, no drinking, only one meal a day, only vegetarian food, no groundnuts, etc.), behavioural requirements (bathing twice daily, visiting the temple daily, no fighting, no eating at the homes of those who are not

also fasting, no visiting homes where there has been a death or a pregnancy), etc.

The festival week is a festive time in the village, with friends and family coming to visit and stay, especially during the last days of the festival. Villagers will have invited friends and relatives who have moved away to stay with them and attend (or, often, participate in) the festival. The temple is busy, with priest and other assistants (many of them being those who will later fulfil vows as part of the festival) attending to the goddess and the various preparations. Those who will participate in the collective rituals of the festival, especially those fulfilling vows, spend additional time in one another's company throughout the festival week. They often worship together at the temple each day, and assist in the preparations for the daily festival events. As those who are fasting are prohibited from visiting the homes of those who are not, they inevitably spend more time socialising with one another. Each night of the festival, non-Dalit men and women dance near the temple (men dance *oyil āṭṭam*, whipping kerchiefs above their heads in synchronised moves, and women dance *kummi aṭi*, clapping and moving in step in a circle).

The major events of the festival happen on the last two days of the week-long event. On the second-to-last day of the festival, the devotees finally fulfil their vows (*nērttikkaṭaṉ*). The day starts with the villagers and their visiting friends and relatives walking over a mile to the nearby river bed. There, the vow-takers prepare to fulfil their vows. A band plays to the crowd, as priests and assistants prepare. The milkpots (*pālkuṭam*) and firepots (*akkiṉiccaṭṭi*) are prepared and the spears and hooks readied. Once everything and everyone are assembled, the priests start the process of piercing the spears and hooks through the cheeks and chests of the devotees taking *kāvaṭi*. The others take up their milkpots and firepots, and the priest and musicians lead the procession to the temple. Carrying their heavy burdens and often becoming possessed as they proceed, the vow-takers advance slowly. Once the village is reached, the procession curves around the village, stopping at other temples along the way, before arriving at the Māriyammaṉ temple. Blocking the way is the bed of hot coals (called the "bed of flowers," the *pūkkuḻi*), across which some (but not all) devotees stride, before finally reaching the temple, seeing the goddess, and depositing their offerings.

Later that evening, the *muḷaippāri* procession occurs. Led by the priest and a god dancer carrying the *caktikarakam* (a pot containing the power (*ṣakti*) of the goddess) non-Dalit Hindu women carry their *muḷaippāri* in a procession encircling the temple. Also part of the procession are others (potentially of any caste) fulfilling a narrow set of vows, most notably those carrying clay images representing some adversity that the goddess helped them overcome: hands or legs for health problems, and children (*tavaḻum piḷḷai*) for the successful birth of a child. After three circumambulations, the *caktikarakam*, *muḷaippāri*, and clay images are deposited at the temple. Yet further acts of devotion follow haphazardly after the *muḷaippāri* procession. Some people place oil lamps made of dough (*māvilakku*) on their bodies in the hopes of curing illnesses, while others carry their children in a cradle hung from sugarcane (*karumpu toṭṭil*) in gratitude for their children and in hopes of their continued health. Others roll prostrated around the temple (*uruṇṭu*), in fulfilment of a vow. (These acts that occur after the *muḷaippāri* procession are distinct, are not considered as part of the collective ritual in analyses). Later

still in the night, and well into the morning, the villagers are entertained by a professional drama troupe. The next morning, the festival is finally completed when the *muḷaippāri* are taken up again and discarded in the temple tank. This marks the end of the festival, and soon after the festival flag is taken down from the temple.

It is important to note that while some forms of worship are open and available to all, some acts are explicitly or tacitly reserved for certain communities. Most importantly, Dalit women (here, members of the Aruntatiyar and Paḷḷar castes) are tacitly prohibited from carrying the *muḷaippāri*, with many people telling me that if they attempted to do so, violence would likely result. However, there are some ways in which men and Dalit women can still participate in the *muḷaippāri* procession: anyone (including Dalits) can carry clay images or the "1000-eyed pot" (*āyiram kaṇ pāṉai*) in the procession, and non-Dalit men can carry special, elaborate *muḷaippāri* and baskets carrying the excess sprouts left over from growing the *muḷaippāri*. Still, thanks to these prohibitions, the participants in the *muḷaippāri* procession are primarily women and non-Dalit. Similarly, while Dalits can fulfil vows and participate in the vow procession, relatively few do. Many more attend the monthly worship.

Participation in the various forms of collective worship at the Māriyammaṉ temple is presented in Tables S2. The number of people who performed particular festival acts in 2012 is presented in Table S3. Note that some individuals performed multiple acts (for example, carrying a milkpot and then walking on the bed of hot coals). Tables S4 and S5 present the demographics of participants (and non-participants) in the monthly worship and annual festival. These tables highlight that some participants are not part of the social support network sample, meaning, generally, that they are not adult Hindu residents of Teṉpaṭṭi. Other participants—primarily residents under the age of 18, relatives who now live elsewhere, and people from neighbouring villages—are not included in the analyses, but are presented here to provide information on full participation and sample coverage. For the monthly worship, out-of-sample participants are primarily residents under the age of 18. For the festival acts, out-of-sample participants are primarily non-residents.

Table S2: The number of adult Hindu residents of Teṉpaṭṭi (who completed the social support survey, N = 248) participating in the monthly worship and the annual festival, and combinations thereof.

			**Festival**			
			No	*Muḷaippāri*	*Vow Procession*	*Both*
			186	34	19	9
**Worship**	*No*	125	107	10	6	2
	Yes	123	79	24	13	7

Table S3: The number of people who participated in each procession in the 2012 festival (total number on the right, number in the social support sample on the left). Note that some individuals carried out multiple acts, so are counted across multiple entries.

	Sample	Full
***Muḷaippāri* Procession**		
Carry sprouts in pot (*muḷaippāri*)	42	176
Carry clay image (*tavaḻum piḷḷai*)	0	2
Grow *muḷaippāri*	2	2
**Vow Procession**		
Carry milkpot (*pālkuṭam*)	6	44
Walk on the bed of hot coals (*pūkkuḻi*)	17	53
Carry firepot (*akkiṉiccaṭṭi*)	4	9
Pierced by spears or hooks (*kāvaṭi*)	4	15
Make firepot (*akkiṉiccaṭṭi*)	2	2
Fast and wear *malai*	11	27

Table S4: Demographic breakdown of participants (and non-participants) for the monthly worship. "Sample" refers to the 248 adult resident Hindus included in the social support network dataset. "Full" refers to the full set of all attendees, including those under 18. Counts for caste and gender; mean and standard deviation for age.

	Monthly Worship		
	Sample		Full
	No	Yes	
**Total**	125	123	138
**Caste**			
Ācāri	8	18	21
Akamuṭaiyār	23	56	66
Aruntatiyar	12	1	1
Kaḷḷar	5	8	9
Kulālar	1	4	4
Nāyakkar	2	1	1
Paḷḷar	57	23	23
Veḷḷāḷar	0	1	1
Yātavar	17	11	12
**Gender**			
Female	61	81	90
Male	64	42	48
**Age**	43.5±17.2	43.7±13.8	40.6±16.0

Table S5: Demographic breakdown of participants (and non-participants) for festival collective rituals. "Sample" refers to the 248 adult resident Hindus included in the social support network dataset. "Full" refers to the full set of all participants in the ritual, including those under 18 and those not resident in the village. Counts for caste and gender; mean and standard deviation for age. (Note that the caste was not known for a few out-of-sample participants).

	Muḷaippāri			Vow Process.		
	Sample		Full	Sample		Full
	No	Yes		No	Yes	
**Total**	205	43	179	220	28	98
**Caste**						
Ācāri	16	10	32	21	5	20
Akamuṭaiyār	61	18	93	64	15	44
Aruntatiyar	13	0	0	13	0	0
Kaḷḷar	7	6	10	12	1	3
Kulālar	4	1	3	3	2	3
Maṟavar	–	–	1	–	–	1
Nāyakkar	2	1	1	3	0	0
Paḷḷar	80	0	2	78	2	10
Paṇṭāram	–	–	1	–	–	1
Veḷḷāḷar	1	0	0	1	0	0
Yātavar	21	7	28	25	3	14
**Gender**						
Female	105	37	154	131	11	42
Male	100	6	25	89	17	56
**Age**	45.4±15.4	35.0±13.5	25.1±13.5	44.5±15.6	36.1±13.9	29.1±13.7

S1.2 Social Support Network

The survey was comprised of two parts, the first part of which was the social support network survey reported here. The second part consisted of further questions asking people about their perceptions and reputational assessments of their peers, and is reported in Ref. [39]. The twelve social support questions were name generators [63], with respondents able to name as many individuals as they liked for each support type. Questions were shaped to allow for easy comparison with other support network data, such as the first question, which asks about discussing important matters, following the classic General Social Survey name generator question [64]. The twelve questions were meant to elicit the range of types of support that individuals might need to call upon. The domains of the questions are similar in scope to other categorizations of social support [65–67] which focus on perceived support and affective or tangible assistance, often received through close ties. Additionally, some questions were included to approximate somewhat the "position generator" [68] and the "resource generator" [69], both of which evoke ties to alters with particular resources and cultural capital. Combined, these twelve support types should give a good sense of a person's social support, including both close, affective bonds and weaker, more distant ties that may be called upon less often, but with greater urgency. An analysis showing the interdependence between the tie types can be found in Ref. [70]. The twelve questions are listed in English and in Tamil in Figure S1.

The survey was conducted in Tamil by graduate students in the Folklore Department at Madurai Kamaraj University, trained by the author in administering the survey. Practically, eight lines were included on the form (filled out by the interviewer), but additional names were written if named by the respondent. After each prompt had been answered, the interviewer copied each unique name to a list for further questions about those individuals. For those individuals who lived in the village (and their immediate families), full demographic data had already been gathered as part of the household survey, so the unique identifier for that person was found and recorded. Interviewees used a census booklet and village map that had details on each household and its members to correctly identify those individuals named by the interviewee. For each household, the name, father's name, gender, age, caste, and current location of each household member was listed. The census booklet included both those presently residing in the village, as well as those relatives who had moved away (primarily residents' adult children and their families). And, kin relations were noted in the booklet with an additional column recording the other households that had immediate relatives to a member of the household. Interviewers could therefore readily establish that they had identified the proper person, and confirm their identification with such questions as "Did you mean the Lakshmi who lives by the school? Whose husband's name is Raju? Whose father's name is Kannan, and whose son is now living in Chennai?" For those not included in the census booklet, the interviewee reported the gender, age, place of residence, employment, and caste of that person, and recorded if they were related to the interviewee or not.

The 248 adult Hindu residents of Teṉpaṭṭi who completed the survey reported an average of 22 people as providing them with support. Those individuals were often named repeatedly

(meaning, a person might be named as both running errands and also lending household items to help the interviewee), so each interviewee actually reported an average of 28 support ties. These nominations combine to a total of 6854 ties, linking together 1516 people through 3809 directed edges (Figure S2). Here, a directed edge from one individual to another represents that the former named the latter as providing her with some form of social support. In the analyses here, the edges are not weighted, meaning that the strength of the edge (the number of ways in which person A helps person B) is not considered. The vast majority of people were named as providing support to someone (people were named as providing support to another an average of 6 or 7 times; 4 of the 248 were not named by other residents of Teṉpaṭṭi, and 6 were not named by other *Hindu* residents of Teṉpaṭṭi as providing support). 44% of the edges (1662 of 3809) were to other adult Hindu residents who completed the survey, meaning that each person named an average of 13 of the other adult Hindu residents as providing them with support. The networks reduced down to the adult Hindu residents who completed the survey is what is used in all analyses, save the final analysis of religious alters. Network summary statistics of the full network and the network reduced to only adult Hindu interviewees are presented in Supplementary Table S6.

Table S6: Network summary statistics for each network under study.

	Full	Adult Hindu Resident
Nodes	1516	248
Edges	3809	1662
Mean Degree	5.025	13.403
Density	0.002	0.027
Reciprocity	0.160	0.366
Transitivity	0.105	0.208
Diameter	8	7
Average Path Length	4.360	3.632
# of Respondents with Out-Degree = 0	1268	0
# of Respondents with In-Degree = 0	6	6

Variable	N	Mean ± SD	Median	Min	Max	# of Levels
Age	248	43.60 ± 15.60	42,43	18	70	–
Gender	248	142 F, 106 M	–	–	–	2
Caste	248	–	–	–	–	9
Years of Education	248	5.28 ± 4.91	5	0	15	–
Ever Committee Member	248	15 Yes, 233 No	–	–	–	2
Household Distance (in meters)	61504	110.33 ± 55.68	109.10	0	317.47	–
Close kin	61504	492 Yes, 61012 No	–	–	–	2

Table S7: Descriptive statistics of the variables used in the models.

Social Survey
சமூகம் சார்ந்த கணக்கெடுப்பு

In our life, each of us in different ways depends on a variety of others for money, work, etc. Now, we will ask questions for their names, to see how many people you are dependent on. Why do you depend on them? Whoever are the helpers for you, only you know.
நம்ம வாழ்க்கையில் ஒவ்வொருவரும் பல்வேறுவிதங்களில் மற்றவர்களை சார்ந்து வாழ்கிறோம் உதாரணமாக பணம், வேலை... இப்போது நாங்கள் கேட்கும் கேள்விகளுக்கு அவர்களுடைய பெயர்களை கூறினால் நீங்கள் எத்தனை பேரை சார்ந்து வாழ்கிறீர்கள்? எதற்காக அவர்களை சார்ந்து இருக்குறீர்கள்? யார்யார் உங்களுக்கு உதவுகிறார்கள் என்புது தெரியும்.

1. If you want to talk about important matters, who do you talk with?
1. நீங்கள் உங்களுடைய முக்கியமான விஷயங்களை பேச விரும்பினால் யாரிடம் பேசுவீர்கள்?

2. If you want daily work [implying daily wage labor] or a new job [implying more permanent employment], who do you approach?
2. உங்களுக்கு அன்றாட வேலை அல்லது புதிய வேலை வேண்டுமென்றால் அணுகக்கூடிய நபர் யார்?

3.Who will amicably help you with physical tasks [meaning, running errands and other chores]?
3. உடல் உழைப்பு மூலம் உங்களுக்கு தோழமையோடு உதவி செய்பவர் யார்?

4. Who do you borrow household items from?
4. உடனடியாக உங்களுக்கு தேவையான வீட்டு உபயோக பொருட்களை யாரிடம் கேட்டுப்பெற்றுகொள்வீர்கள்?

5. If you suddenly need a small amount of money for something, whom would you ask for it from?
5. அவசரதேவைக்கு கொஞ்ச பணம் தேவையென்றால் யாரிடம் கேட்பீர்கள்?

6. If you need a lot of money, whom would you ask for it from [meaning, a loan]?
6. உங்களுக்கு அதிக பணம் தேவையென்றால் யாரிடம் கேட்பீர்கள்?

7. If you have to go to work and need someone to watch your child, who would you give them to?
7. நீங்கள் வேலைக்கு போகும்போது குழந்தையை பார்த்துக்கொள்ள வேண்டுமென்றால் யாரிடம் விட்டு செல்வீர்கள்?

8.If you had to spend a lot of time talking with someone, who would you like to talk with?
8. நீங்கள் அதிக நேரம் ஒருவரோடு பேசிக்கொண்டிருக்க விரும்பினால், யாரோடு பேச வேண்டும் என்று நினைப்பீர்கள்?

9. If any problem happens, who are the people who will help you?
9. பிரச்சனை எதுவும் நடந்தால் உங்களுக்கு உதவும் நபர்கள் யார்யார்?

10. Who do you know well in a "high position" [e.g., government officials, police, lawyers, teachers, etc.]
10. உங்களுக்கு பழக்கமானவர்கள், உயர்ந்த பதவியில் உள்ளவர்கள் யார்?

11. Who are your very close friends or relatives?
11. உங்களுக்கு மிகவும் நெருக்கமான நண்பர்கள் மட்டும் உறவினர்கள் யார்யார்?

12. Who are the people who give you advice?
12. உங்களுக்கு ஆலோசனை வழங்கும் நபர் யார்?

Figure S1: Text of the social support network survey in English and Tamil.

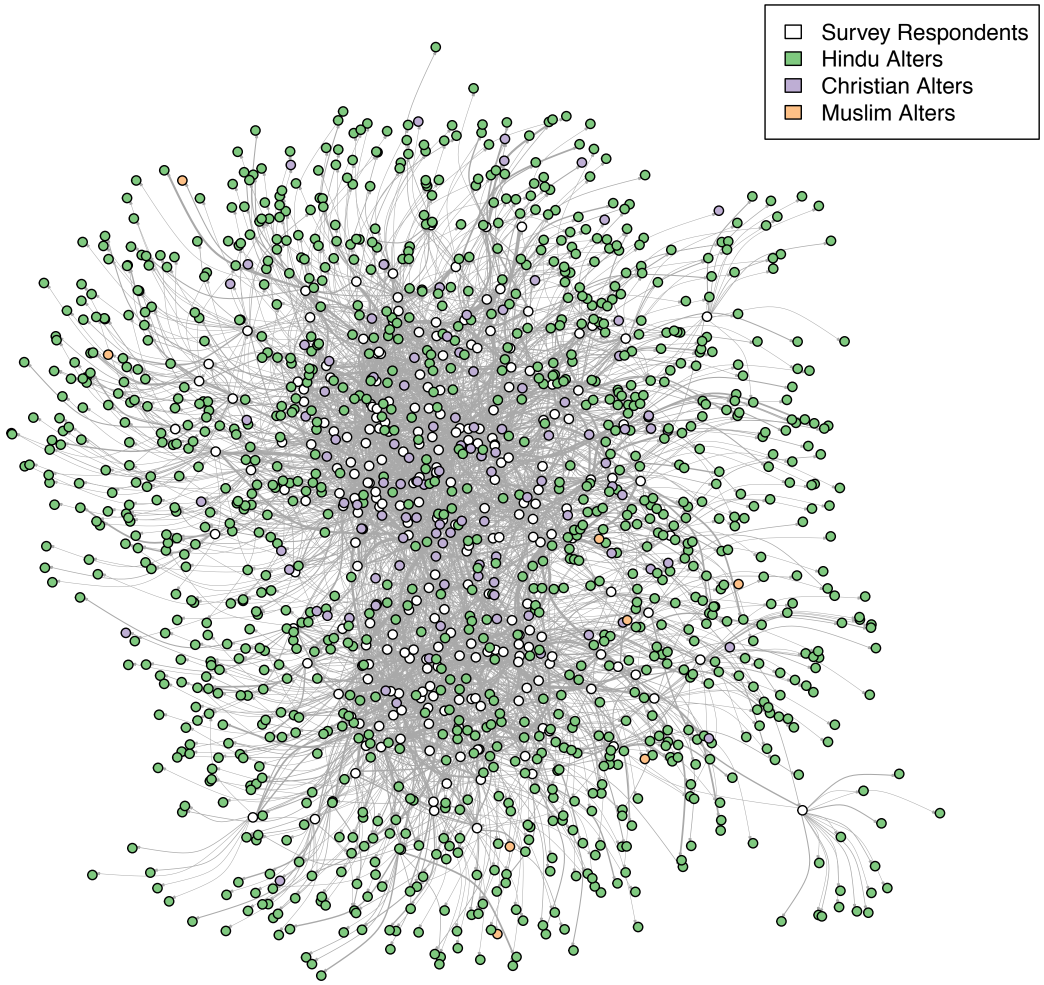

Figure S2: The network showing all ties reported by the 248 interviewees. The 248 adult Hindu resident interviewees are colored white, and the remaining 1268 alters named by them are coloured by religion.

S2 Exponential Random Graph Models

Table S8: Description of the variables used in the exponential random graph models. Node terms reflect the impact of a person's (node's) attributes on the probability of a support tie (an edge). Edge terms capture the effect of some relationship between every two set of individuals (each dyad) on the probability of a tie (e.g., whether they are of the same gender or the physical distance between their households). "In" refers to terms that affect incoming ties (meaning, people naming that person as providing them with support). Terms without the "in" qualifier include effects of the variable on both incoming (people naming that person) and outgoing ties (that person naming others). Covariates are numeric predictors while factors are categorical.

Variable	Term type	Description
Age	Node covariate	The individual's age.
Gender Homophily	Edge factor	Whether two individuals have the same gender.
Gender	Node in-factor	The individual's gender.
Close Kin	Edge factor	Whether two individuals are related as parent/child, siblings, or spouses.
Education Years Difference	Edge covariate	The absolute difference in the total number of years of education.
Ever Committee Member	Node covariate	Whether or not a person has ever held a position on either the informal village committee or the panchayat.
Distance between Households	Edge covariate	The distance (in 10 meter units) between individuals' houses.
Caste Homophily	Edge factor	Whether two individuals are of the same caste.
Caste	Node factor	The caste membership of the individual.
Reciprocity	Edge factor	The effect of a supportive tie in one direction on the probability of a reciprocal tie.
Shared Edge Partners	Edge factor	Geometrically-weighted edge-wise shared partners (GWESP).
Shared Dyad Partners	Edge factor	Geometrically-weighted dyad-wise shared partners (GWDSP).
Monthly Worship Co-Participation	Node match	Whether two individuals both participated in the monthly worship, with only a term for matched participation, not matched non-participation.
Vow Procession Co-Participation	Node match	Whether two individuals both participated in the festival vow procession, with only a term for matched participation, not matched non-participation.
Muḷaippāri Co-Participation	Node match	Whether two individuals both participated in the monthly worship, with only a term for matched participation, not matched non-participation.
Regular worship	Node in-factor	Whether the person worships at a temple at least once a week [39, 40].
Possession	Node in-factor	Whether the person becomes possessed on a semi-regular basis [39, 40].
Weighted Public Ritual Tally	Node in-covariate	The tally, weighted by difficulty and monetary cost, of public ritual acts carried out across the previous year [39, 40].

Table S9: Exponential random graph model predicting the log-odds of a tie in the Teṉpaṭṭi Hindu adult social support network, including a measure of co-participation in the Māriyammaṉ festival in 2012.

	Estimate	Std. Error	Odds Ratio	p-value
Edges	-3.292	0.161	0.037	<.0001
**Covariates**				
Age	-0.003	0.001	0.997	0.0016
Same Gender (No = 0)	0.594	0.048	1.811	<.0001
Gender (Female = 0)	-0.041	0.026	0.960	0.1220
Close Kin (No = 0)	2.259	0.103	9.578	<.0001
Education Years Difference	-0.044	0.006	0.957	<.0001
Ever Committee Member (No = 0)	0.542	0.069	1.720	<.0001
Distance (10 m units)	-0.073	0.005	0.930	<.0001
Same Caste (No = 0)	0.667	0.056	1.949	<.0001
Caste: Ācāri		[reference category]		
Caste: Akamuṭaiyār	0.027	0.039	1.027	0.4976
Caste: Aruntatiyar	0.060	0.072	1.062	0.4035
Caste: Veḷḷāḷar	0.805	0.228	2.236	0.0004
Caste: Yātavar	0.094	0.057	1.098	0.1000
Caste: Kaḷḷar	0.263	0.067	1.301	0.0001
Caste: Kulālar	0.143	0.109	1.154	0.1872
Caste: Nāyakkar	0.570	0.183	1.769	0.0018
Caste: Paḷḷar	-0.107	0.039	0.899	0.0061
**Religiosity**				
Festival Vow Procession Co-Participation	0.282	0.158	1.326	0.0739
Muḷaippāri Co-Participation	0.130	0.111	1.138	0.2449
**Structural Terms**				
Mutual	1.102	0.115	3.010	<.0001
GWESP ($\alpha = 0.4$)	1.019	0.043	2.772	<.0001
GWDSP ($\alpha = 0.4$)	-0.090	0.007	0.914	<.0001

Null deviance = 84919, $df = 61256$
Residual deviance = 9992, $df = 61234$
Log-likelihood = -4996.16
AIC = 10036.32
BIC = 10234.82

Table S10: Exponential random graph model predicting the log-odds of a tie in the Teṉpaṭṭi Hindu adult social support network, including a measure of co-participation in the Māriyammaṉ monthly worship.

	Estimate	Std. Error	Odds Ratio	p-value
Edges	-3.367	0.161	0.034	<.0001
**Covariates**				
Age	-0.003	0.001	0.997	0.0011
Same Gender (No = 0)	0.599	0.048	1.820	<.0001
Gender (Female = 0)	-0.016	0.027	0.984	0.5441
Close Kin (No = 0)	2.291	0.098	9.883	<.0001
Education Years Difference	-0.043	0.006	0.958	<.0001
Ever Committee Member (No = 0)	0.511	0.071	1.666	<.0001
Distance (10 m units)	-0.071	0.005	0.931	<.0001
Same Caste (No = 0)	0.663	0.055	1.941	<.0001
Caste: Ācāri		[reference category]		
Caste: Akamuṭaiyār	0.009	0.040	1.009	0.8186
Caste: Aruntatiyar	0.121	0.076	1.129	0.1118
Caste: Veḷḷāḷar	0.727	0.233	2.069	0.0018
Caste: Yātavar	0.129	0.057	1.138	0.0243
Caste: Kaḷḷar	0.285	0.066	1.329	<.0001
Caste: Kulālar	0.124	0.115	1.132	0.2802
Caste: Nāyakkar	0.625	0.186	1.869	0.0008
Caste: Paḷḷar	-0.065	0.041	0.937	0.1153
**Religiosity**				
Monthly Worship Co-Participation	0.274	0.048	1.315	<.0001
**Structural Terms**				
Mutual	1.095	0.115	2.988	<.0001
GWESP ($\alpha = 0.4$)	1.003	0.043	2.725	<.0001
GWDSP ($\alpha = 0.4$)	-0.094	0.008	0.910	<.0001
Null deviance = 84919, df = 61256				
Residual deviance = 9965, df = 61235				
Log-likelihood = -4982.71				
AIC = 10007.42				
BIC = 10196.90				

Table S11: Exponential random graph model predicting the log-odds of a tie in the Teṉpaṭṭi Hindu adult social support network, including all measures of co-participation and direct terms for religious action.

	Estimate	Std. Error	Odds Ratio	p-value
Edges	-3.563	0.170	0.028	<.0001
**Covariates**				
Age	-0.003	0.001	0.997	0.0024
Same Gender (No = 0)	0.604	0.049	1.830	<.0001
Gender (Female = 0)	-0.016	0.028	0.984	0.5721
Close Kin (No = 0)	2.301	0.101	9.984	<.0001
Education Years Difference	-0.041	0.006	0.960	<.0001
Ever Committee Member (No = 0)	0.449	0.071	1.567	<.0001
Distance (10 m units)	-0.071	0.005	0.931	<.0001
Same Caste (No = 0)	0.668	0.055	1.951	<.0001
Caste: Ācāri		[reference category]		
Caste: Akamuṭaiyār	0.047	0.041	1.048	0.2486
Caste: Aruntatiyar	0.168	0.076	1.183	0.0274
Caste: Veḷḷāḷar	0.757	0.231	2.132	0.0010
Caste: Yātavar	0.163	0.059	1.178	0.0054
Caste: Kaḷḷar	0.299	0.069	1.348	<.0001
Caste: Kulālar	0.128	0.113	1.136	0.2591
Caste: Nāyakkar	0.632	0.192	1.881	0.0010
Caste: Paḷḷar	-0.052	0.043	0.949	0.2235
**Religiosity**				
Monthly Worship Co-Participation	0.214	0.058	1.239	0.0002
Festival Vow Procession Co-Participation	0.209	0.168	1.232	0.2151
Muḷaippāri Co-Participation	0.098	0.112	1.103	0.3780
Regular Worship (No = 0)	0.090	0.057	1.094	0.1162
Possession (No = 0)	-0.241	0.081	0.786	0.0030
Weighted Public Ritual Tally	0.018	0.005	1.019	0.0002
**Structural Terms**				
Mutual	1.123	0.116	3.074	<.0001
GWESP ($\alpha = 0.4$)	0.984	0.043	2.676	<.0001
GWDSP ($\alpha = 0.4$)	-0.095	0.007	0.909	<.0001
Null deviance = 84919, df = 61256				
Residual deviance = 9942, df = 61230				
Log-likelihood = −4971.154				
AIC = 9994.31				
BIC = 10228.90				

Table S12: Stepwise exponential random graph models predicting the log-odds of a tie in the Teṉpaṭṭi Hindu adult social support network, showing model coefficients with standard errors in parentheses.

	**Model 1**	**Model 2**	**Model 3**	**Model 4**
Edges	-3.253^{***}	-3.367^{***}	-3.292^{***}	-3.563^{***}
	(0.155)	(0.161)	(0.161)	(0.170)
**Covariates**				
Age	-0.003^{***}	-0.003^{**}	-0.003^{**}	-0.003^{**}
	(0.001)	(0.001)	(0.001)	(0.001)
Same Gender (No = 0)	0.598^{***}	0.599^{***}	0.594^{***}	0.604^{***}
	(0.049)	(0.048)	(0.048)	(0.049)
Gender (Female = 0)	$-0.044^{\dagger}$	-0.016	-0.041	-0.016
	(0.026)	(0.027)	(0.026)	(0.028)
Close Kin (No = 0)	2.259^{***}	2.291^{***}	2.259^{***}	2.301^{***}
	(0.100)	(0.098)	(0.103)	(0.101)
Education Years Difference	-0.044^{***}	-0.043^{***}	-0.044^{***}	-0.041^{***}
	(0.006)	(0.006)	(0.006)	(0.006)
Ever Committee Member (No = 0)	0.550^{***}	0.511^{***}	0.542^{***}	0.449^{***}
	(0.068)	(0.071)	(0.069)	(0.071)
Distance (10 m units)	-0.073^{***}	-0.071^{***}	-0.073^{***}	-0.071^{***}
	(0.005)	(0.005)	(0.005)	(0.005)
Same Caste (No = 0)	0.671^{***}	0.663^{***}	0.667^{***}	0.668^{***}
	(0.055)	(0.055)	(0.056)	(0.055)
Caste: Ācāri		[reference category]		
Caste: Akamuṭaiyār	0.020	0.009	0.027	0.047
Caste: Aruntatiyar	0.043	0.121	0.060	0.168^{*}
Caste: Veḷḷāḷar	0.786^{***}	0.727^{**}	0.805^{***}	0.757^{**}
Caste: Yātavar	0.082	0.129^{*}	$0.094^{\dagger}$	0.163^{**}
Caste: Kaḷḷar	0.256^{***}	0.285^{***}	0.263^{***}	0.299^{***}
Caste: Kulālar	0.153	0.124	0.143	0.128
Caste: Nāyakkar	0.558^{**}	0.625^{***}	0.570^{**}	0.632^{**}
Caste: Paḷḷar	-0.124^{**}	-0.065	-0.107^{**}	-0.052
**Religiosity**				
Monthly Worship Co-Participation		0.274^{***}		0.214^{***}
		(0.048)		(0.058)
Festival Vow Procession Co-Participation			$0.282^{\dagger}$	0.209
			(0.158)	(0.168)
Muḷaippāri Co-Participation			0.130	0.098
			(0.111)	(0.112)
Regular Worship (No = 0)				0.090
				(0.057)
Possession (No = 0)				-0.241^{**}
				(0.081)
Weighted Public Ritual Tally				0.018^{***}
				(0.005)
**Structural Terms**				
Mutual	1.104^{***}	1.095^{***}	1.102^{***}	1.123^{***}
	(0.116)	(0.115)	(0.115)	(0.116)
GWESP ($\alpha = 0.4$)	1.022^{***}	1.003^{***}	1.019^{***}	0.984^{***}
	(0.042)	(0.043)	(0.043)	(0.043)
GWDSP ($\alpha = 0.4$)	-0.089^{***}	-0.094^{***}	-0.090^{***}	-0.095^{***}
	(0.008)	(0.008)	(0.007)	(0.007)
AIC	10037.372	10007.422	10036.323	9994.308
BIC	10217.828	10196.901	10234.825	10228.902
Log Likelihood	-4998.686	-4982.711	-4996.161	-4971.154

$^{***}p < 0.001$, $^{**}p < 0.01$, $^{*}p < 0.05$, $^{\dagger}p < 0.10$

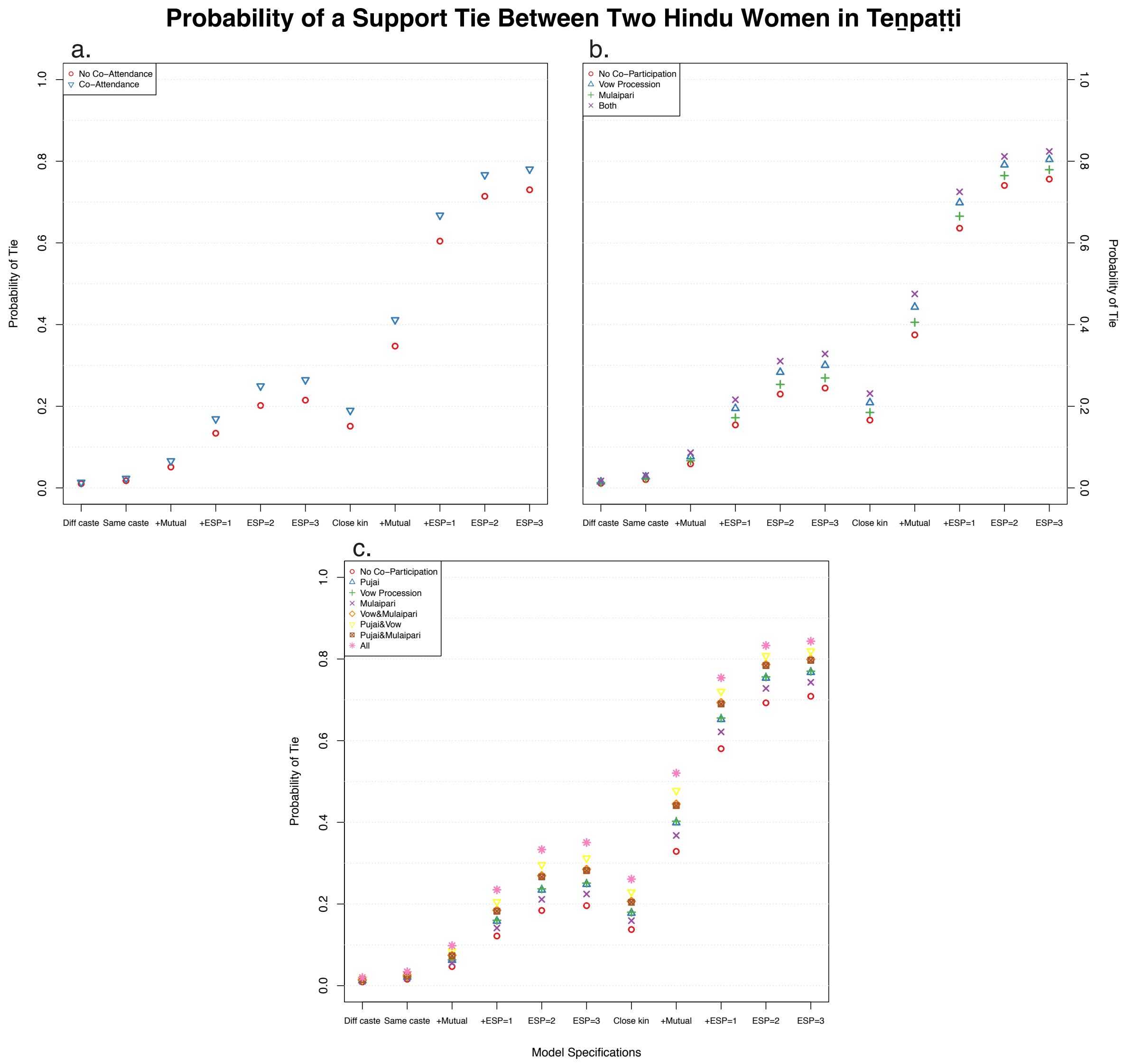

Figure S3: The probability of a tie for the Teṉpaṭṭi Hindu support network, given various combinations of node- and dyad-level attributes and (a) whether the dyad attends the Māriyammaṉ *pauṟṇami pūjai* together (from the model presented in Table S10), (b) whether the dyad co-participates in the Māriyammaṉ festival together (from the model presented in Table S9), and (c) combinations therein (from the model presented in Table S11). The leftmost points (gathered under "Diff caste") represent the predicted probability of each support tie for two unrelated Hindu women, one of the Yātavar caste and one of the Akamuṭaiyār caste, of the same age (42) and educational attainment, with no friends in common, no existing relationship, neither having served on any political committee, with 100 meters between their houses. The next set starts with the same base, except now they are of the same caste (both Akamuṭaiyār). The next additionally adds that they already have one mutual tie (i.e., the likelihood of a reciprocal tie). The next adds that they have one common support partner (with their tie having no further impact on other edgewise shared partners), then two common partners (with their tie having no further impact on other edgewise shared partners), then three (with their tie having no further impact on other edgewise shared partners). Next, we revert back to no common ties, but now the two Akamuṭaiyār women are close kin. Then we additionally add again one tie between them, one common partner, two common partners, three common partners. For these fitted probabilities, the change statistic associated with the dyad-wise shared partner term (GWDSP) is the median value for edges that correspond to the hypothetical edge-wise partner change, as observed in the actual network.

S3 Network Cohesion Measures

Excess edges is a measure of community coherence that is conceptually related to density. Differences in density within and between groups show where a network's edges have accumulated, relative to a completely random network. However, it may be that differences in density of ties between religious participants are due not to their co-participation but instead due to differences in the numbers of support ties themselves, i.e. individuals' degrees. To address this problem, excess edges adds one additional layer of sophistication by comparing to a graph whose nodes have exactly the same degrees, but whose edges are otherwise placed entirely at random, a widely used model that is referred to as the "configuration model" [71]. In other words, excess edges measures excess density while controlling for in- and out-degrees. The excess edges quantity Q is related to a measure of community structure in networks called modularity [72], and is computed in Eq. (S1):

$$Q_w = \sum_{ij} \left(A_{ij} - \frac{k_i^{\text{out}} k_j^{\text{in}}}{m} \right) \delta(g_i, w)\delta(g_j, w) \ , \tag{S1}$$

where $\delta(x, y)$ is the Kronecker delta [$\delta(x, y) = 1$ only when $x = y$; otherwise $\delta(x, y) = 0$], g_i is the group associated with individual i, A_{ij} is the adjacency matrix, m is the total number of edges in the network, and k_i^{in} and k_i^{out} are the number of in- and out-degrees of individual i, respectively. Note that the summed term in the equation above corresponds intuitively to the name "excess edges": it counts the number of edges in the real network that exceed the expected number under a fixed-degree random network.

Table S13: Measures of the cohesion (excess edges, density, transitivity, and reciprocity) of the network subgraphs for each type of co-participation. The subgraphs of monthly worshippers at the Māriyamman̲ temple (N = 123) and participants in the 2012 Māriyamman̲ festival (N = 64) are calculated in reference to the Ten̲paṭṭi Hindu subgraph (N = 248). The vow procession participant subgraph (N = 28) is calculated in reference to two subgraphs: first, the Ten̲paṭṭi Hindu subgraph, and second the Māriyamman̲ festival participant subgraph. First, the "raw" *p*-values are reported (i.e., the probability of getting a measure that is as great or greater than the actual measure from the observed subgraph), and then *p*-values adjusted with the Bonferroni correction for multiple comparisons.

	**Excess Edges**		**Density**		**Transitivity**		**Reciprocity**	
	Value	*p*-value	Value	*p*-value	Value	*p*-value	Value	*p*-value
**"Raw" *p*-values**								
All Hindu			0.027		0.208		0.366	
Monthly Worship	95.047	<.0001	0.042	<.0001	0.208	0.4841	0.413	0.0376
Annual Festival	50.045	<.0001	0.045	<.0001	0.272	0.1038	0.429	0.1369
Vow Procession (ref: Hindu)	13.108	0.0032	0.058	0.0005	0.231	0.3513	0.364	0.4779
Vow Procession (ref: Fest)	2.956	0.1610	0.058	0.0774	0.231	0.5529	0.364	0.7206
***p*-values with Bonferroni correction**								
All Hindu			0.027		0.208		0.366	
Monthly Worship	95.047	<.0001	0.042	<.0001	0.208	1.0000	0.413	0.1592
Annual Festival	50.045	<.0001	0.045	0.0004	0.272	0.3868	0.429	0.5248
Vow Procession (ref: Hindu)	13.108	0.0128	0.058	0.0020	0.231	1.0000	0.364	1.0000
Vow Procession (ref: Fest)	2.956	0.6320	0.058	0.3252	0.231	1.0000	0.364	1.0000

S4 Religious Alters

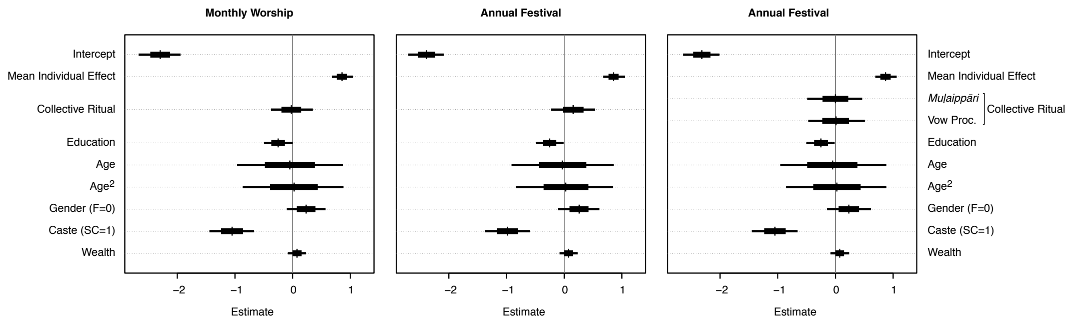

Figure S4: Summary of posterior distributions of parameters in the logistic regression models presented in Table S14, modelling individuals' number of ties with people of other religious denominations. Where possible, the variables have been centred and rescaled. Thick lines show the 65% and thin lines show the 95% probability intervals.

Table S14: Logistic regressions of the probability that an alter will be of a different religion for Hindu residents of Teṉpaṭṭi who do or do not participate in the Māriyammaṉ temple monthly worship or the annual festival, showing the estimate, the standard deviation of the estimate, and the 95% highest posterior density intervals (HPDI).

	Estimate	SD	HPDI Low	HPDI High
**Monthly Worship**				
Intercept	-2.296	0.187	-2.671	-1.944
Mean Individual Effect	0.855	0.095	0.683	1.049
Monthly Worship (No = 0)	-0.024	0.184	-0.373	0.350
Education	-0.252	0.126	-0.496	0.000
Age	-0.049	0.464	-0.963	0.875
Age^2	0.024	0.439	-0.867	0.881
Gender (Female = 0)	0.232	0.174	-0.104	0.570
Caste (BC = 0)	-1.050	0.200	-1.443	-0.669
Wealth	0.075	0.082	-0.085	0.234
**General Festival Participation**				
Intercept	-2.385	0.158	-2.703	-2.088
Mean Individual Effect	0.857	0.094	0.678	1.051
Festival (No = 0)	0.155	0.194	-0.227	0.532
Education	-0.251	0.124	-0.491	-0.010
Age	-0.033	0.450	-0.914	0.858
Age^2	0.027	0.424	-0.840	0.848
Gender (Female = 0)	0.259	0.179	-0.105	0.611
Caste (BC = 0)	-0.985	0.196	-1.373	-0.594
Wealth	0.076	0.080	-0.081	0.233
**Festival Events**				
Intercept	-2.315	0.161	-2.643	-2.009
Mean Individual Effect	0.868	0.095	0.692	1.062
Muḷaippāri (No = 0)	-0.001	0.243	-0.490	0.465
Vow Procession (No = 0)	0.011	0.248	-0.471	0.510
Education	-0.251	0.126	-0.504	-0.013
Age	-0.047	0.466	-0.955	0.883
Age^2	0.026	0.440	-0.858	0.884
Gender (Female = 0)	0.233	0.192	-0.148	0.616
Caste (BC = 0)	-1.048	0.199	-1.451	-0.656
Wealth	0.075	0.082	-0.085	0.239

References

[63] Marsden PV. 1990 Network data and measurement. *Annual Review of Sociology* **16**, 435–463.

[64] Marsden PV. 1987 Core discussion networks of Americans. *American Sociological Review* **52**, 122–131.

[65] Barrera M, Ainlay SL. 1983 The structure of social support: A conceptual and empirical analysis. *Journal of Community Psychology* **11**, 133–143.

[66] Wellman B, Wortley S. 1990 Different strokes from different folks: community ties and social support. *The American Journal of Sociology* **96**, 558-588.

[67] Uchino BN. 2004 Social support and physical health understanding the health consequences of relationships. New Haven, CT: Yale University Press.

[68] Lin N, Dumin M. 1986 Access to occupations through social ties. *Social Networks* **8**, 365–385.

[69] Van der Gaag M, Snijders TAB. 2005 The Resource Generator: social capital quantification with concrete items. *Social Networks* **27**, 1–29.

[70] De Bacco C, Power EA, Larremore DB, Moore C. 2017 Community detection, link prediction, and layer interdependence in multilayer networks. *Physical Review E* **95**, 042317.

[71] Fosdick BK, Larremore DB, Nishimura J, Ugander J. 2018 Configuring random graph models with fixed degree sequences. *SIAM Review.* (http://arxiv.org/abs/1608.00607)

[72] Girvan M, Newman MEJ. 2002 Community structure in social and biological networks. *Proceedings of the National Academy of Sciences* **99**, 7821–7826.
